# The application of time-to-event analysis in machine learning prognostic models

**DOI:** 10.1186/s12967-024-04909-1

**Published:** 2024-02-12

**Authors:** Zi-He Peng, Zhi-Xin Huang, Juan-Hua Tian, Tie Chong, Zhao-Lun Li

**Affiliations:** 1 Department of Urology, The Second Affiliated Hospital of Xi’an Jiaotong University, Xi’an, 710004 China; 2 Health Science Center, Xi’an Jiaotong University, Xi’an, 710061 China

**To the editor,**

Artificial intelligence (AI), with its ambition to emulate human intelligence within machines, has emerged as a transformative force across various domains. Within the realm of modern medicine, the digitization of practices such as electronic medical records has ushered in new opportunities for the integration of machine learning (ML) methodologies. These innovations find application in diverse contexts, from AI-assisted pathology assessments to the ML-driven analysis of qualitative interviews and medical records, unearthing intricate themes and underlying patterns. In the clinical sphere, ML frequently directs its focus toward enhancing predictive capabilities, harnessing the potential of commonplace and readily accessible variables to refine prognostic accuracy. It’s worth noting that while many ML analyses focus on classification issues and the creation of diagnostic models, in the medical field, the prevalent approach involves using survival analysis to develop prognostic models.

Survival analysis, an intricate statistical method, is designed to unravel the intricate correlations between covariates and the temporal occurrences of events. Unlike conventional classification paradigms, survival analysis confronts the intricacies engendered by partially observed data, often stemming from censoring. Within the realm of clinical inquiries, patient records manifest in distinct categories: those that remain uncensored, thereby divulging precise event timings, and those that exist as right-censored, withholding event timings beyond the study’s temporal scope. This distinctive attribute mandates the utilization of specialized models adept at accommodating the complexities inherent to such data structures, thus emerging as a pivotal facet within the realm of survival analysis methodology.

Regrettably, it is observed that many recently published articles have erred by simplistically transforming outcomes into categorical variables and utilizing ML classification techniques to formulate prognostic models [1, 2]. These endeavors have been undertaken without due consideration for the impact of censored data on the model’s fidelity. A systematic review uncovered that among 11 studies crafting 24 models for survival outcomes, merely ten models explicitly took into account censored observations, of which seven were built upon the framework of Cox regression [3]. This implies that only three ML models are considered censored data. These studies usually exclude patients who survive but for shorter than a specific date, after which several date-specific models are built (e.g., 3-year, 5-year). To employ a straightforward analogy, where a traditional statistical model, specifically Cox proportional hazard regression, should have been adopted to construct prognostic models predicting survival at 3-year and 5-year intervals, logistic regression was employed to create two categorical models. As underscored by PROBAST (Prediction model risk of Bias ASsessment Tool), the exclusion of censored participants through simplistic logistic regression models is deemed unsuitable [4]. The utilization of an erroneous logistic regression methodology results in a selected dataset containing fewer individuals lacking the outcome, thus introducing bias into predicted risks due to the overrepresentation of those with the outcome [4]. The time-to-event analysis provides an effective means of addressing these censored observations. Contrary to the notion that there are no ML algorithm packages for conducting survival analysis, there is indeed a Python module named “scikit-survival” designed for this purpose. It is developed on top of scikit-learn and can be found at https://scikit-survival.readthedocs.io/en/latest/index.html (accessed on October 30, 2023) [5]. This module enables the incorporation of survival analysis within the capabilities of scikit-learn. We highly advise employing “scikit-survival” for the development of ML prognostic models.

When developing ML prognostic models, it is strongly advised to employ survival analysis techniques such as “scikit-survival” to appropriately handle censored observations. Simply excluding or categorizing censored cases using logistic regression is inappropriate and introduces bias. Overall, ignoring censoring and using inaccurate evaluation metrics can severely compromise the validity of machine learning-based prognostic models. Careful consideration of censoring and time-to-event analysis principles is warranted.

## Data Availability

Not applicable.

## Abbreviations

AI: Artificial intelligence
ML: Machine learning
PROBAST: Prediction model risk of Bias ASsessment Tool

## References

[1] Karabacak M, Jagtiani P, Carrasquilla A, Germano IM, Margetis K (2023). Prognosis individualized: survival predictions for WHO grade II and III gliomas with a machine learning-based web application. NPJ Digit Med.

[2] Li C, Liu M, Zhang Y, Wang Y, Li J, Sun S, Liu X, Wu H, Feng C, Yao P (2023). Novel models by machine learning to predict prognosis of breast cancer brain metastases. J Transl Med.

[3] Dhiman P, Ma J, Andaur Navarro CL, Speich B, Bullock G, Damen JAA, Hooft L, Kirtley S, Riley RD, Van Calster B (2022). Methodological conduct of prognostic prediction models developed using machine learning in oncology: a systematic review. BMC Med Res Methodol.

[4] Moons KGM, Wolff RF, Riley RD, Whiting PF, Westwood M, Collins GS, Reitsma JB, Kleijnen J, Mallett S (2019). PROBAST: a tool to assess risk of bias and applicability of prediction model studies: explanation and elaboration. Ann Intern Med.

[5] Polsterl S (2020). scikit-survival: a library for Time-to-event analysis built on top of scikit-learn. J Mach Learn Res.

